# A Modified Technique Reduced Operative Time of Laparoendoscopic Rendezvous Endoscopic Retrograde Cholangiopancreatography Combined with Laparoscopic Cholecystectomy for Concomitant Gallstone and Common Bile Ductal Stone

**DOI:** 10.1155/2014/861295

**Published:** 2014-06-15

**Authors:** Wei Liu, Qunwei Wang, Jing Xiao, Liying Zhao, Jiangsheng Huang, Zhaohui Tan, Pengfei Li

**Affiliations:** Minimal Invasive Surgery Center, Department of General Surgery, Second Xiangya Hospital, Central South University, 139 Renmin Road, Changsha City, Hunan Province 410011, China

## Abstract

Laparoendoscopic rendezvous (LERV) endoscopic retrograde cholangiopancreatography (ERCP) and laparoscopic cholecystectomy (LC+ERCP/LERV) are considered an optimal approach for concomitant gallstones and common bile duct stones. The rendezvous technique is essential for the success of procedure. We applied two different LERV techniques, traditional technique and modified technique, in 60 consecutive cases from January 2011 to November 2012. 32 cases who underwent modified technique (group 1) from February 2012 to November 2012 were retrospectively compared to 28 cases (group 2) who underwent traditional technique from January 2011 to January 2012. There was no significant difference between two groups with respect to preoperative demographic features. Although the difference was not statistically significant, the procedure was successfully performed in 31 cases (96.9%) in group 1 and 24 cases (86.2%) in group 2. The mean operative time and time of endoscopic part were 82.6 ± 19.6 min and 26.5 ± 5.99 min in group 1 which were significantly shorter than those in group 2 (118.0 ± 23.1 min and 58.7 ± 13.3 min, resp.). There was no postoperative pancreatitis and mortality in both groups. The mean hospital stay, blood loss, incidence of complications, and residual stone were of no difference in both groups. This study proved that this modified technique can effectively reduce the operative time and time of endoscopic part of LC+ERCP/LERV compared with traditional technique.

## 1. Introduction

Common bile ductal stone is concomitant with gallstone in approximately 10% of the gallstone patients [1, 2]. Laparoscopic cholecystectomy (LC) with simultaneous intraoperative endoscopic retrograde cholangiopancreatography (ERCP) using laparoendoscopic rendezvous (LERV) technique was reported to be an optimal approach for concomitant gallstone and common bile ductal stone because it offers significant advantage over the traditional two-stage methods such as LC combined with preoperative or postoperative ERCP [3–7]. Rendezvous technique, which is essential for the success of procedure, involves a sequence of maneuver and collaboration between surgeon and endoscopist. Although several kinds of techniques have been reported [5, 8, 9], there was no comparative study investigating different techniques of LERV. After utilization of traditional technique of LERV on early cases, we have developed a modified technique of LERV which demonstrated favorable surgical results by comparing to traditional technique. In this report, we present our single center study of two different techniques of LERV.

## 2. Material and Methods

60 consecutive cases of gallstone with concomitant common bile ductal stone were operated with simultaneous LC and intraoperative ERCP with LERV technique (LC+ERCP/LERV) by the same team of surgeon and endoscopist (who is also a surgeon) from January 2011 to November 2012. 32 cases who underwent modified procedure (group 1; *n* = 32) from February 2012 to November 2012 were retrospectively compared to 28 cases who underwent traditional procedure (group 2; *n* = 28) from January 2011 to January 2012.

All the patients were preoperatively diagnosed with concomitant gallstone and common bile ductal stone by magnetic resonance cholangiopancreatography (MRCP). Under general anesthesia, modified technique and traditional technique of LC+ERCP/LERV were conducted on patients of group 1 and group 2, respectively, which were described in detail as follows.

### 2.1. Procedure of the Modified Technique

**Figure 1:**
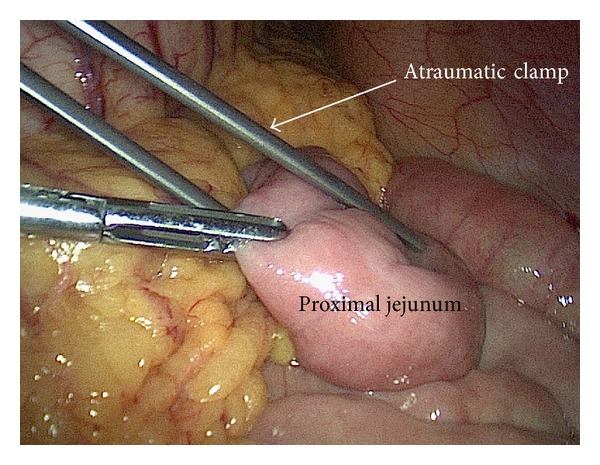
Application of the atraumatic clamp on jejunum.

**Figure 2:**
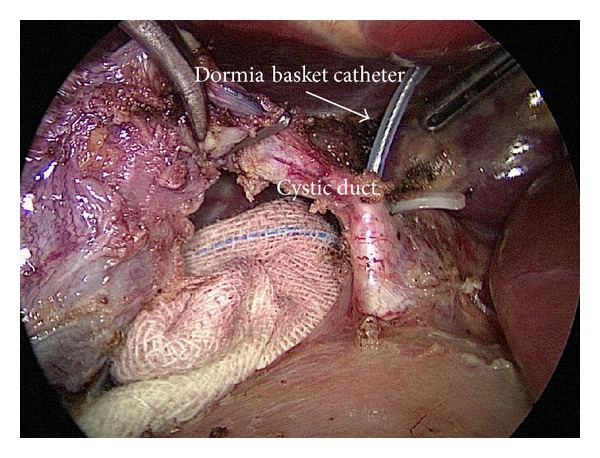
Insertion of Dormia basket catheter into cystic duct.

**Figure 3:**
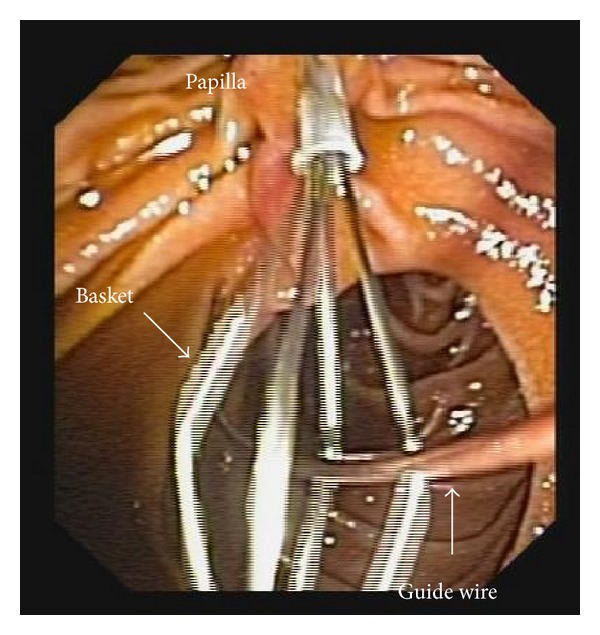
Introducing the tip of guide wire into the basket.

**Figure 4:**
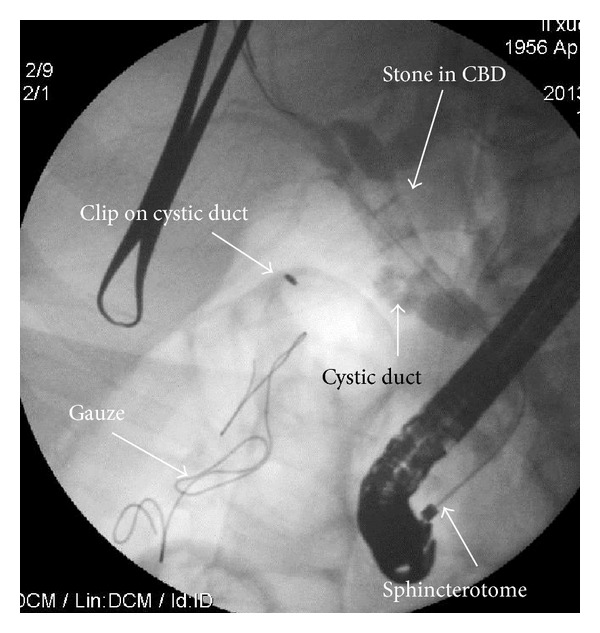
Stones revealed in cholangiography after selective bile duct cannulation.

The patient was in supine position under general anesthesia. Three trocars were placed as in routine LC, a 10 mm trocar for 30-degree endoscope at umbilicus, a 12 mm trocar at midline below xiphoid for dissection and clip applier, a 5 mm trocar at right midclavicular line under costal margin for retracting grasper, and an extra 5 mm trocar at right anterior axillary line below costal margin sometimes required for better retraction. LC was conducted using slightly anti-Trendelenburg and left tilting position; cystic artery was first dissected and divided after clipping; the gallbladder was dissected off the liver with only cystic duct kept intact; then a clip was applied on cystic duct close to Hartmann's pouch. The operating table was adjusted to slight Trendelenburg position; an atraumatic clamp 6 cm in length was applied on jejunum about 10 cm distal to Treitz ligament to temporarily block the passage of air into distal part of small bowel (Figure 1). Then a small opening was created by hook on cystic duct below the preapplied clip. A 7 French catheter was then inserted to perform intraoperative cholangiogram using a C-arm fluoroscopy. Once the presence of CBD stone was confirmed by cholangiogram, a 7 French Dormia basket catheter was inserted into CBD through the opening on cystic duct (Figure 2). With adjustment of the angle and repeated attempts, the basket catheter could be pushed through the papilla entering duodenum. At this time, the endoscopist inserted the endoscope (TJF-260, Olympus) to duodenum until the papilla was visualized, overseeing the Dormia basket catheter passing through the papilla. Under the endoscopic monitoring, a sphincterotome loaded with guide wire was introduced through endoscope to approach the opened basket; the guide wire was advanced until its hydrophilic tip was trapped into basket (Figure 3). The laparoscopic surgeon then closed the basket to grasp the guide wire and pulled back the basket catheter so that the guide wire would follow the catheter and enter CBD; the sphincterotome was then advanced over the guide wire by the endoscopist to achieve elective CBD cannulation. At this point of time, the surgeon opened the basket and let the endoscopist retreating guide wire; then the surgeon removed the basket catheter and clipped the cyst duct distal to the opening to avoid bile leakage during the subsequent process. The sphincterotomy and balloon dilatation was then performed accordingly, followed by the CBD stone clearance using basket or balloon catheter under fluoroscopic guidance as in routine ERCP (Figure 4). After completion of the endoscopic part of the procedure, the endoscope was withdrawn after aspirating the air and fluid in duodenum and stomach. The surgeon then divided the cystic duct between clips into complete cholecystectomy, followed by removal of the clamp on jejunum. After hemostasis and irrigation, drainage was placed near Winslow orifice.

### 2.2. Procedure of the Traditional Technique

Procedure was the same as the modified procedure except the section of rendezvous technique which was also described in other reports [5, 10]; a guide wire was used to pass through the cyst duct and manipulated by the surgeon to pass through duodenal papilla; then the hydrophilic tip of guide wire was gasped with snare by the endoscopist; with collaborative pushing by the surgeon, the guide wire was pulled out of endoscope with snare. Then the sphincterotome was introduced along the guide wire to achieve bile duct cannulation, which was followed by the according stone-extraction operation as in routine ERCP.

### 2.3. Statistical Analysis

Continuous variables were presented as mean ± SD; categorical variables are presented as percentages. Comparison of continuous variables such as hospital stay, blood loss, time of endoscopic part, and operative time between the 2 groups was performed by unpaired* t*-test. Categorical variables such as rate of conversion and morbidity were compared by chi-squared test. All tests of statistical significance were 2-tailed and were considered to be significant at a level of 0.05. Statistical analyses were carried out using SPSS statistical software (version 10.0; SPSS, Chicago, Ill).

## 3. Results

The demographic and biochemical findings of both groups were demonstrated in Table 1. There was no significant difference between the two groups with regard to the age, BMI, level of serum bilirubin, level of gamma-glutamyl transferase, diameter of common bile duct, diameter of largest stone, and the incidence of multiple stone.

The surgical results of both groups were summarized in Table 2. Although the difference was not statistically significant, the rate of conversion to common bile ductal exploration due to failure of LC+ERCP/LERV was higher in group 2 than in group 1 (13.8% versus 3.1%). The failed case in group 1 was found to be stone incarceration at distal end of CBD, leading to the inability of guide wire to pass through duodenal papilla. The guide wire failed to pass through cystic duct in 2 cases and duodenal papilla in another 2 cases in group 2. The mean operative time for endoscopic part (from the insertion to the withdrawal of endoscope) was significantly longer in group 2 than in group 1 (58.7 min versus 26.5 min). The operative time was significantly shorter in group 1 than that in group 2 (82.6 min versus 118 min) between the two groups. The incidence of complication was not significantly different between the two groups; there was no postoperative pancreatitis in both groups, whereas 1 (3.1%) case in group 1 and 2 cases in group 2 demonstrated elevated level of serum amylase at postoperative day 1 and reduced to normal range at postoperative day 4 without any symptom and physical sign. There was 1 (3.1%) case in group 1 presented with postoperative fluid collection around gallbladder fossa which was treated by percutaneous drainage. There was no mortality in both groups. The mean hospital stay was 5.2 days in group 1 and 5.5 days in group 2, showing no significant difference.

Follow-up was obtained in all cases at 6 months postoperatively; there was no incidence of stone recurrence according to transabdominal ultrasound findings which were conducted during the follow-up.

## 4. Discussion

LC+ERCP/LERV has been proved to be an optimal approach for concomitant gallstones and CBD stones [6, 7, 10], but the traditional technique of LERV, based on our own experience, was found to have some drawbacks: (1) it was difficult in some cases to manipulate the guide wire when passing it through cystic duct and duodenal papilla; (2) it could be time consuming during the reciprocal maneuver of pushing and pulling guide wire out of endoscope.

The failure of LERV has been reported in previous studies. Tzovaras et al. [10] reported that LERV were converted to traditional intraoperative ERCP in 6 cases of LERV group (*n* = 50) due to the failure of advancing guide wire through cystic duct into duodenum. In a perspective study of LERV in which traditional technique was used, El-Geidie [11] reported 3 failed cases of LERV because of the inability of passing guide wire through cystic duct. We agree with his opinion that in some patients advancing the guide wire through a spiral tortuous cystic duct is tedious and time consuming. We also found that it is quite difficult in some cases to control the direction of the guide wire and advance it though duodenal papilla. Tommasi et al. [5] reported in a study of LERV with traditional technique (*n* = 96) that guide wire failed to be introduced through cyst duct in 2 cases and duodenal papilla in 6 cases; we encountered failure of advancing guide wire through cystic duct in 2 cases and duodenal papilla in another 2 cases in group 2 of our study in which traditional technique was applied. Although the difference of conversion rate is not statistically significant in our study (3.1% in group 1 versus 13.8% in group 2), there was only 1 failed case in group 1 which was in consistence with the fact that we found it easier to manipulate the Dormia basket catheter than guide wire to advance through cystic duct and duodenal papilla.

The bowel insufflation and bile leakage through cystic duct during procedure were considered drawbacks of intraoperative ERCP [11], which could be worse if the duration of ERCP prolonged. Therefore, it is meaningful to reduce the time of both whole procedure and endoscopic part. We initially utilized the traditional technique as described in other reports of LC+ERCP/LERV [9, 10, 12, 13], of which guide wire was inserted and advanced through duodenal papilla then pulled out through entire endoscope channel by a snare; the sphincterotome was then introduced over guide wire to achieve bile duct cannulation. After being applied in our early cases as in group 2 of this study, we found that this traditional technique requires excessive maneuvers between reciprocal pushing and pulling guide wire by surgeon and endoscopist which result in prolonged operative time. Tekin et al. [9] reported in their study comparing LERV and laparoscopic antegrade sphincterotomy that LERV are associated with prolonged time because more maneuver such as introducing and pulling guide wire was needed in the procedure. In the modified technique as adopted in group 1 of our study, a Dormia basket catheter was used for advancing through papilla and gasping the guide wire which was introduced simultaneously with sphincterotome; the bile ductal cannulation was simply achieved by pulling back the guide wire and simultaneous advancing of sphincterotome. With traditional technique, however, the sphincterotome could not be introduced until major part of guide wire was extracted out of endoscope by snare which required more maneuver of pushing and pulling guide wire by surgeon and endoscopist. In addition, we also noted that the traditional technique of LERV required at least two surgeons to handle the guide wire as one fixes the cystic duct with laparoscopic forceps and the other advances the guide wire through trocar. From our experience, there were several advantages demonstrated by the modified technique which was utilized in group 1 of our study: (1) it was quicker and simpler to achieve bile ductal cannulation; (2) because the surgeon did not need to advance the guide wire, one surgeon is adequate to complete the whole LERV procedure in most cases. According to the retrospective analysis in this study, the time of endoscopic part (TOE) was significantly shorter in the group with modified technique comparing with that in the group with traditional technique, suggesting that the modified technique could save the time spending on LERV and ERCP. The overall operative time was unsurprisingly shorter in group 1 than that in group 2 with a reduction of 36 min, proving that the overall operative time could also be saved by the modified technique. Although overall operative time was analyzed in most studies [3, 5, 9, 11], TOE during LC+ERCP/LERV was rarely investigated except that Tzovaras [10] reported TOE of 32 min in his study of LERV with traditional technique. Despite that TOE was longer in group 2 of our study than in Tzoravas's study (58 min versus 32 min) in which traditional technique was used, a significant reduction of TOE from traditional technique to modified technique (58 min versus 26 min) was demonstrated in our single center study. Although there was no data support, we think the shortened TOE might have some value in reducing risk of bile leakage during LERV and bowel insufflation during ERCP.

Since LERV ruled out the risk of pancreatic ductal injection and cannulation [6], no incidence of postoperative pancreatitis was observed in our study. However, postoperative asymptomatic hyperamylasemia presented in 1 case of group 1 and 2 cases in group 2 in our study, of which we thought was a result from balloon dilatation (12 mm) for retrieval of large stones, as similar findings was described in other reports [6, 10, 14]. The incidence of complications was of no significant difference between the two groups, suggesting that there is no increased risk associated with the modified technique. Because the tip of Dormia basket catheter was blunt and the passage of catheter through papilla was monitored by endoscope, there was no Dormia basket catheter associated iatrogenic injury observed in our study.

The rate of bile ductal stone clearance was reported higher in the method of LERV than in sequential approach (ERCP before LC) [3], in which traditional technique of LERV was applied. In our study, there was no residual stone presented in both groups, indicating that bile ductal stone clearance was not affected by different techniques of LERV. Although there was no comparative analysis from the perspective of cost effectiveness in this study, similar cost might be indicated by the insignificantly different hospital stay and complication rate between the two groups. With regard to the logistic cost, the extra consumption of a Dormia basket catheter in modified technique was partially balanced by the sparing of a snare catheter which was needed in traditional technique.

## 5. Conclusion

The modified technique of LERV can reduce TOE and operative time. Because of the retrospective nature of this study, this modified technique need to be further investigated by prospective randomized trial.

## Figures and Tables

**Table 1:** Demographic of patients of concomitant gallstones and common bile duct stones.

	Group 1	Group 2	*P* value
Total case number	**32**	**28**	
Male/female (*n*)	15/17	15/13	
Mean age (years)	49.3 ± 19.5 (19–86)	51.59 ± 20.8 (23–88)	0.357
Mean BMI (kg/m^2^)	23.1 ± 3.7 (18.4–31)	22.52 ± 3.22 (18.2–31.4)	0.173
Mean total bilirubin (*μ*mol/L)	29.6 ± 23.5 (10.7–112.3)	34.0 ± 29.6 (9.1–238.4)	0.470
Mean *γ*-GT (*μ*/L)	194.7 ± 269.5 (12.9–1102.5)	221.5 ± 341.4 (13.2–1543.9)	0.503
Mean DCBD (mm)	13.9 ± 3.4 (8–20)	12.5 ± 3.3 (8–20)	0.623
Mean DLS (mm)	6.2 ± 2.2 (3–12)	6.3 ± 2.49 (3–15)	0.712
Multiple stones (*n*)	9 (28.1%)	8 (28.6%)	0.597

DCBD: Diameter of common bile duct

DLS: Diameter of largest stone

*γ*-GT: gamma-glutamyl transferase.

**Table 2:** Surgical result of laparoendoscopic rendezvous ERCP combined with laparoscopic cholecystectomy.

	Group 1	Group 2	*P* value
Total case numbers	**32**	**28**	
Conversion to LCBDE (*n*)	1 (3.1%)	4 (13.8%)	0.182

For the unconverted cases	Group 1	Group 2	
Mean operative time (min)	82.6 ± 19.6 (58–156)	118.0 ± 23.1 (85–185)	0.038
Blood loss (mL)	45.0 ± 10.8 (30–80)	44.3 ± 18.2 (20–85)	0.187
Mean time of endoscopic part (min)	26.5 ± 5.99 (15–40)	58.7 ± 13.3 (35–90)	0.017
Mean hospital stay (day)	5.2 ± 0.8 (4–9)	5.5 ± 1.1 (5–9)	0.109
Postoperative morbidity (*n*)	2 (6.2%)	2 (7.1%)	0.641
Hyperamylasemia (*n*)	1 (3.1%)	2 (7.1%)	
Abdominal fluid collection (*n*)	1 (3.1%)		
Residual stone (*n*)	0 (0%)	0 (0%)	

LCBDE: laparoscopic common bile duct exploration.
